# Effect of whitening toothpaste on surface roughness and microhardness of human teeth: a systematic review and meta-analysis

**DOI:** 10.12688/f1000research.76180.3

**Published:** 2022-03-08

**Authors:** Navodita Jamwal, Ashwini Rao, Ramya Shenoy, Mithun Pai, Aparna KS, Avinash BR

**Affiliations:** 1 Public Health Dentistry, Manipal College of Dental Sciences, Mangalore, Manipal Academy of Higher Education, Manipal, India, Mangalore, Karnataka, 575001, India

**Keywords:** dental enamel, in-vitro study, meta-analysis, micro hardness, surface roughness, systematic review, whitening toothpaste

## Abstract

**Background**: Whitening toothpastes exert a whitening effect on teeth through higher surface cleaning effectiveness resulting from the abrasive properties of the paste or specific chemical components. This systematic review and meta-analysis was conceptualized to examine the relationship between whitening toothpastes and surface roughness as well as microhardness of human teeth and to clarify the evidence base available around this relationship by conducting a systematic review and meta-analysis of studies in this topic area, looking at *in vitro* randomized control trials.

**Methods**: Criteria for including studies in the review were done based on population, intervention, comparison, outcomes and study and studies were identified from electronic databases. Covidence® was used for data screening and data extraction. The CONSORT tool was used for checking relevant content and methodology used in each of the papers reviewed. Systematic review was done followed by meta-analysis, using Review Manager.

**Results**: A total of 125 articles were obtained on key word search. After duplicate removal and title screening, 17 articles were eligible for full text review. Finally, 7 studies were included for systematic review and meta-analysis was conducted on 4 studies. The forest plot for surface roughness showed that that the meta-analytic effect was statistically significant with surface roughness value being higher in the intervention group. The forest plot for microhardness showed that the meta-analytic effect was statistically significant with the microhardness value being lesser in the intervention group.

**Conclusions**: Although whitening toothpastes typically can lighten tooth color by about one or two shades, there is some evidence to show that these toothpastes also affect the mineral content of teeth by increasing surface roughness and reducing microhardness. More evidence and further research are needed to identify the type of whitening agent which will whiten the tooth effectively while maintaining the integrity of the tooth structure.

## Introduction

“Tooth whitening is a conservative and effective method to lighten discolored teeth and has been practiced in dentistry for many centuries”. ^1^ Management of discolored teeth, was earlier done by tooth whitening material in the form of oxalic acid, chlorine, ammonia and hydrogen peroxide. ^2^ Whitening of teeth can be done professionally in the dental practice by scaling and polishing, bleaching or by using prosthetic crowns; it can also be done at home, by the individual themselves, using an over the counter whitening toothpaste. ^1^ The role of a whitening toothpaste is to remove unwanted surface deposits and stains with minimal effect on the tooth structure. ^3^

Whitening toothpastes exert their action either because of the abrasive properties of the paste or because of specific chemical components, such as silica, aluminum oxide, sodium bicarbonate, carbamide peroxide and hydrogen peroxide or a combination of these. ^4^ Although all toothpastes contain abrasives, whitening toothpastes often contain a higher amount of harder abrasives. ^5^ Bleaching compounds like calcium and magnesium peroxide and sodium percarbonate have also been used in whitening toothpastes. Other whitening agents that have been used are surfactants, colorants, enzymes and polyaspartate. ^6^^,^^7^ Presently some toothpastes have also started using activated charcoal as a whitening agent because it has the capacity of adsorbing pigments and stains. ^1^ Studies have shown that whitening toothpastes can bring about changes to the surface roughness and microhardness of teeth. ^8^^,^^9^

## Rationale

The extraordinary mechanical properties of a tooth with respect to hardness and fracture toughness is due to the chemical and structural interaction between the inorganic hydroxyapatite and the organic protein matrix. ^1^ Studies have reported structural damage to enamel surface prisms and increased tooth sensitivity during professional teeth whitening in the dental clinic. ^10^^–^^12^ However, conflicting results have been reported with respect to the clinical efficiency of home use whitening toothpastes, with many studies reporting very little clinically significant effect on tooth whitening. ^13^^–^^15^ Most whitening toothpastes contain abrasives of different sizes and shapes and as the size of the abrasive particles increases, the abrasiveness of a toothpaste also increases leading to increased surface roughness and reduced microhardness of the enamel.

This systematic review and meta-analysis was conceptualized with the hope that this understanding might help in managing toothpaste formulations to bring about tooth whitening without affecting surface roughness and microhardness. The findings would have clinical implications as well as implications for research.

## Objective

To assess the effect of whitening toothpastes on the surface roughness and microhardness of human teeth by identifying all relevant literature, evaluating it systematically and synthesizing the data to integrate the findings.

## Focus question

We attempted to answer the following question:

Do whitening toothpastes affect the surface roughness and microhardness of human teeth?

## Methods

### Eligibility criteria

*Inclusion criteria*

Criteria for including studies in the review were done based on **PICOS**,

i. **P**opulation, or participants and conditions of interest: Extracted human teeth
ii. **I**nterventions or exposures: Brushing with whitening toothpaste
iii. **C**omparisons or control groups: With at least one comparison group
iv. **O**utcomes of interest: Surface roughness and microhardness
v. **S**tudy designs: *In vitro* RCT studies

*Exclusion criteria*

It was decided to exclude studies in languages other than English and studies where abstracts or full texts were not available.

### Information sources

Studies were identified from the electronic databases of Scopus, Embase (EMBASE, RRID:SCR_001650), PubMed (PubMed Central, RRID:SCR_004166), Springer Link, Web of Science (Clarivate Analytics, RRID:SCR_017657) and Cochrane Central Register of Controlled Trials (CENTRAL) (Cochrane Library, RRID:SCR_013000) between February and October 2021.

### Search strategy

Search used the following key terms with the Boolean ‘OR’ operator: “dental enamel” OR “microhardness” OR “surface roughness” OR “ *in vitro* study” OR “whitening toothpaste” sort by: relevance, Filters: English.

The selection of articles was completed by two authors (Jamwal N and Rao A) using papers published in the electronic databases, as assessed by the eligibility criteria. Reference checking and hand searching of articles was also done.

### Data collection process

Reviewer number 1 (NJ) and reviewer number 2 (AR) screened the articles independently. Any disagreements were resolved by a third author (RS). Covidence ^®^ (Veritas Health Innovation, Melbourne, Australia) (RRID:SCR_016485) was used for data screening and data extraction. The CONSORT tool was used for checking relevant content and methodology used in each of the papers reviewed. Systematic review was done followed by meta-analysis, using Review Manager (RevMan version 5.4.1) (RRID:SCR_003581).

### Data items

Data was sought for two outcomes namely, surface roughness and microhardness of extracted human teeth, with at least two time points i.e., before and after intervention with a whitening toothpaste. In cases where there were more than one post intervention time points, the final time point was considered. The Covidence data extraction template was customized for this systematic review.

### Risk of bias assessment

The quality of the articles was assessed using the Revised Cochrane risk-of-bias tool for randomized trials (RoB 2), which is structured into five domains, risk of bias arising from the randomization process, due to deviations from intended interventions, due to missing outcome data, bias in measurement of the outcome and bias in selection of the reported result.

The Covidence quality assessment template was customized for this study. Reviewer number 1 (NJ) and reviewer number 2 (AR) reviewed the quality of the articles independently. In case of any disagreements, discussions were held to come to a consensus.

### Data synthesis

Data was analyzed with the random effects meta-analyses model for continuous data, for the two outcomes of surface roughness and microhardness, using Review Manager (RevMan 5.4.1) (RevMan, RRID:SCR_003581). Forest plots were constructed in Review manager for the two outcomes of surface roughness and microhardness. Publication bias was assessed by constructing funnel plots using Review Manager.

## Results

### Study selection

**Figure 1.**
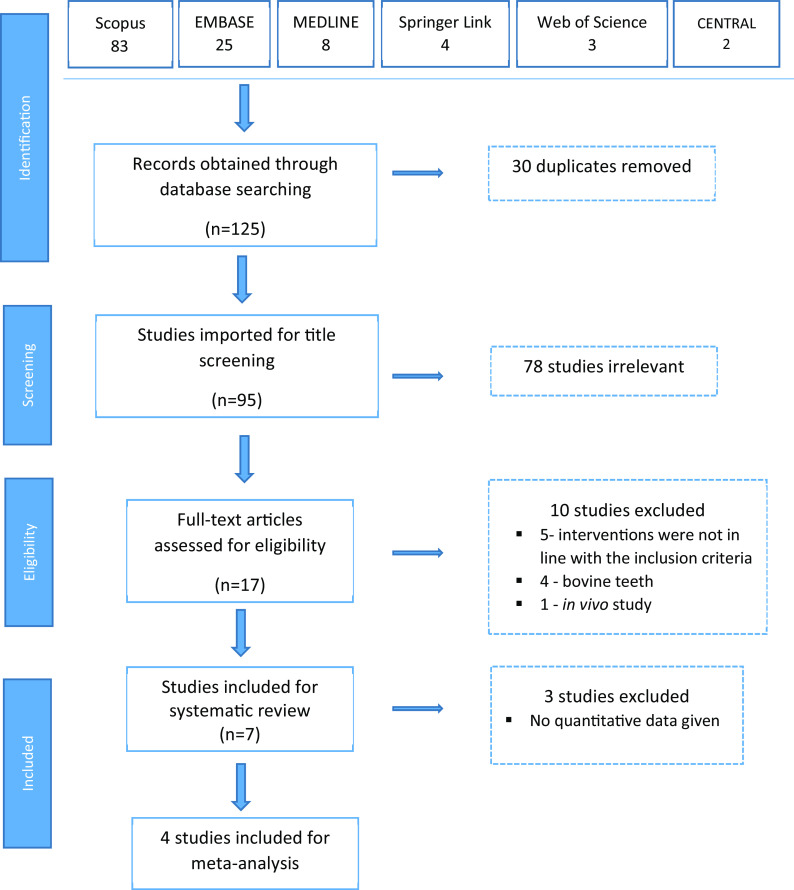
Flow chart of steps in literature search.

A total of 125 articles were obtained on the initial key word search ( Table 1), out of which 83 were from Scopus, 25 from Embase, 8 from PubMed/MEDLINE, 4 from Springer Link, 3 from Web of Science and 2 from the Cochrane Library. When the 30 duplicates were removed, we selected 95 articles for level 1 title screening. After title screening, 78 studies were found to be irrelevant, and 17 articles were eligible for full text review. During the full text review, 10 studies were excluded; 5 because the interventions were not in line with the inclusion criteria, 4 because the studies used bovine teeth and one because it was an *in vivo* study. Finally, 7 studies were included for systematic review and meta-analysis was conducted on 4 studies ( Figure 1).

**Table 1.** Characteristics of included studies.

Author, year	Control group	Intervention group/whitening ingredient	Sample size	Brushing duration	Type of outcome measures	Key conclusions
Bolay 2012	• Control group brushed with water without dentifrice • Colgate Total (control)	• Natural White	8 in each group	Exposed to 20,000 brush strokes	Surface roughness and microhardness	Toothbrushing with whitening dentifrice increased surface roughness values but had no effect on hardness values.
Feitosa 2013	• Colgate Total Advanced Clean (control)	• Colgate Total Advanced Whitening • Colgate Whitening Oxygen Bubbles	12 in each group	Brushed for 20,000 cycles to simulate 10 hours	Surface roughness	Whitening dentifrices increased the surface roughness of enamel.
Rahardjo 2015	• Pepsodent Regular (control)	• Pepsodent Whitening • Formula Sparkling White	20 in each group	Brushed for 840 seconds to simulate 3 months	Surface roughness and microhardness	Tooth brushing with whitening toothpaste for a prolonged time increased enamel roughness and decreased enamel microhardness.
Shamel 2019	• Close Up (control for close up white now) • Sensodyne (control for Sensodyne true white) • Colgate (control for Colgate optic white) • Control with no tooth paste application	• Close up White now • Sensodyne True White • Colgate Optic White	10 in each group	Brushed for 420 minutes, simulating 4 weeks	Surface roughness	Blue covarine containing toothpastes produced less surface abrasion in comparison with blue covarine-free toothpastes.
Alpan Lektemur 2020	• Control group (water brushing only)	• Sensodyne True White • Splat Special Blackwood • Colgate Optic White • Signal White Now • Ipana 3D White • Paradontax Whitening	20 in each group	Brushed for 5 seconds per day for 30 days.	Surface roughness	The two whitening toothpastes i.e., Splat Special Blackwood and Colgate Optic White, reduced enamel roughness, whereas no significant changes were seen with the other whitening toothpastes.
Maden 2021	• Colgate MaxFresh (control)	• İpana White Power	30 in each group	Brushed for 2 min twice a day for 1 week	Surface roughness and microhardness	Ipana White Power toothpaste increased surface roughness and reduced microhardness.
Vural 2021	• Colgate Total (control)	• Body Kingdom • Curaprox Black is White • Colgate Optic White	12 in each group	Hundred and sixty-eight cycles of brushings to simulate 12-weeks.	Surface roughness and microhardness	Except for the Curaprox Black is white, all other toothpastes showed increased surface roughness, while microhardness was not affected in any of the groups.

### Characteristics of included studies

The characteristics of included studies are summarized in Table 1. All selected studies were published between 2012 and 2021. The studies used control and comparison groups to evaluate the effects of whitening toothpaste on surface roughness and microhardness of human teeth *in vitro.* Some studies used brushing without toothpaste as the control group ^16^^,^^17^ while others used regular toothpastes as controls. ^8^^,^^9^^,^^18^^-^^20^ The intervention consisted of a variety of whitening toothpastes like Natural White, ^16^ Colgate Total Advanced Whitening, Colgate Whitening Oxygen Bubbles, ^18^ Pepsodent Whitening, Formula Sparkling White, ^8^ Close up White now, Sensodyne True White, Colgate Optic White, ^19^ Sensodyne True White, Splat Special Blackwood, Colgate Optic White, Signal White Now, Ipana 3D White, Paradontax Whitening, ^17^ İpana White Power ^9^ and Body Kingdom, Curaprox Black is White, Colgate Optic White. ^20^

The sample sizes varied from 8 per group to 30 per group with four studies having both surface roughness and microhardness as the outcomes and three studies having only one outcome of surface roughness.

### Risk of bias in studies

**Figure 2.**
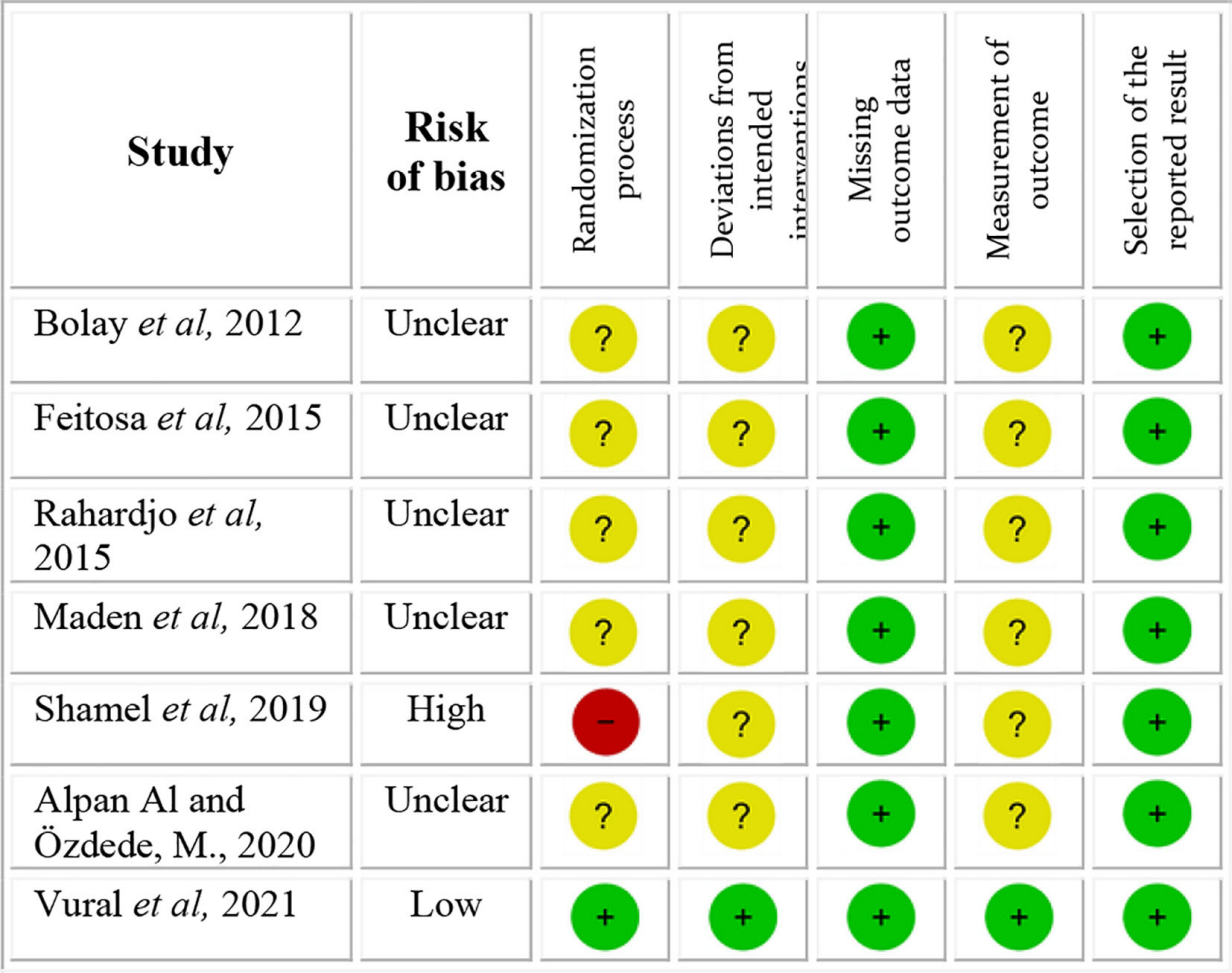
Risk of bias scale.

The quality of the 7 articles was assessed using the Revised Cochrane risk-of-bias tool for randomized trials (RoB 2), and is shown in Figure 2. One study showed a high risk of bias and one a low risk of bias. All other 5 studies showed unclear risk of bias. The main concerns were with respect to the risk of bias arising from the randomization process, risk of bias due to deviations from intended interventions and risk of bias in measurement of the outcome.

The study ^19^ with high risk of bias not only gave no information on the random sequence allocation, but also reported the presence of unsolved baseline differences between intervention groups suggesting a problem with the randomization process. All studies with unclear risk of bias showed lack of information with respect to the random allocation sequence, blinding of people delivering the intervention and blinding of the outcome assessors.

### Meta-analysis

We used random-effects meta-analyses model assuming that underlying effects follow a normal distribution. Among the 7 studies that were included for systematic review, quantitative data was inappropriate for 3 studies ^9^^,^^17^^,^^19^ and one ^19^ showed high risk of bias and therefore these studies could not be included, and meta-analysis was conducted using the remaining 4 studies. Data from the 4 studies ^8^^,^^16^^,^^18^^,^^20^ selected for meta-analysis were analyzed to create forest plots displaying weights and confidence intervals. Separate forest plots were created for the two outcomes of surface roughness and microhardness.

*Forest plot for surface roughness*

**Figure 3.**
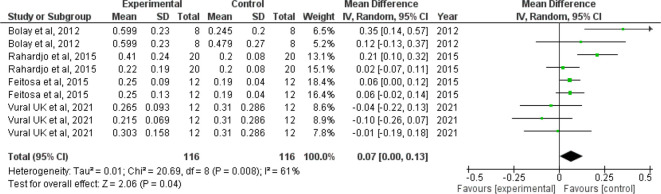
Forest plot comparing the surface roughness of enamel of controls with those brushed with whitening toothpaste.

The I ^2^ value of 61% indicates moderate heterogeneity. The confidence interval of the combined effect size (diamond) does not include zero and is on the right hand side, indicating that the meta-analytic effect is statistically significant and favoring the control (confidence level of 95%; p-value is less than.05). This shows that the surface roughness value is lesser in the control group when compared with the intervention group. The corresponding Z value is 2.06 and the p value is 0.04 ( Figure 3).

*Forest plot for microhardness*

**Figure 4.**
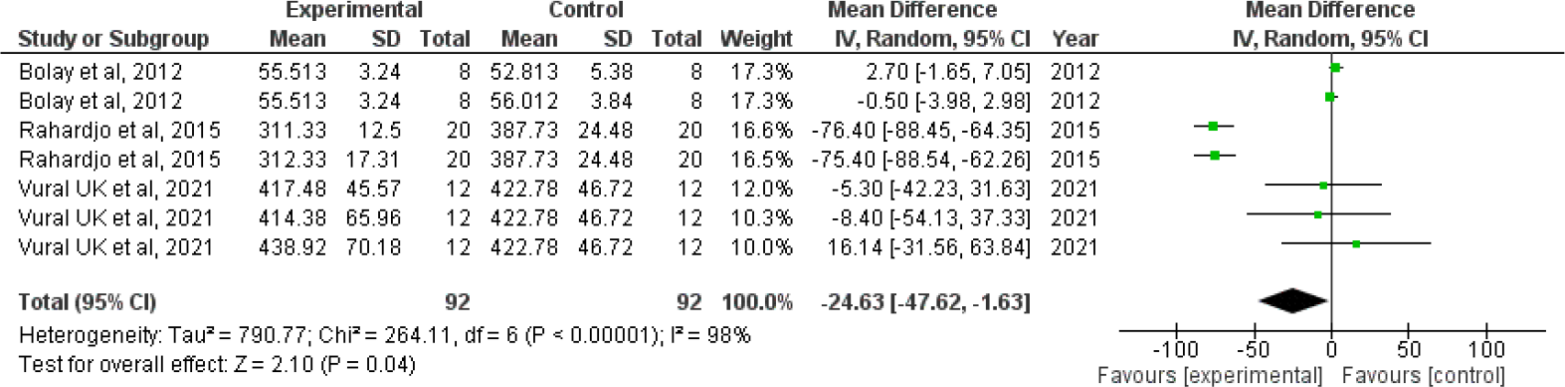
Forest plot comparing the microhardness of enamel of controls with those brushed with whitening toothpaste.

Since I ^2^ value is 98% showing high heterogeneity, the results need to be interpreted with caution. The confidence interval of the combined effect size (diamond) does not include zero and is on the left hand side, indicating that the meta-analytic effect is statistically significant and favoring the experimental group (confidence level of 95%; p-value is less than 0.05). This shows that the microhardness value is lesser in the intervention group when compared with the control group. The corresponding Z value is 2.10 and the p value is 0.04 ( Figure 4).

*Funnel plot:*

**Figure 5.**
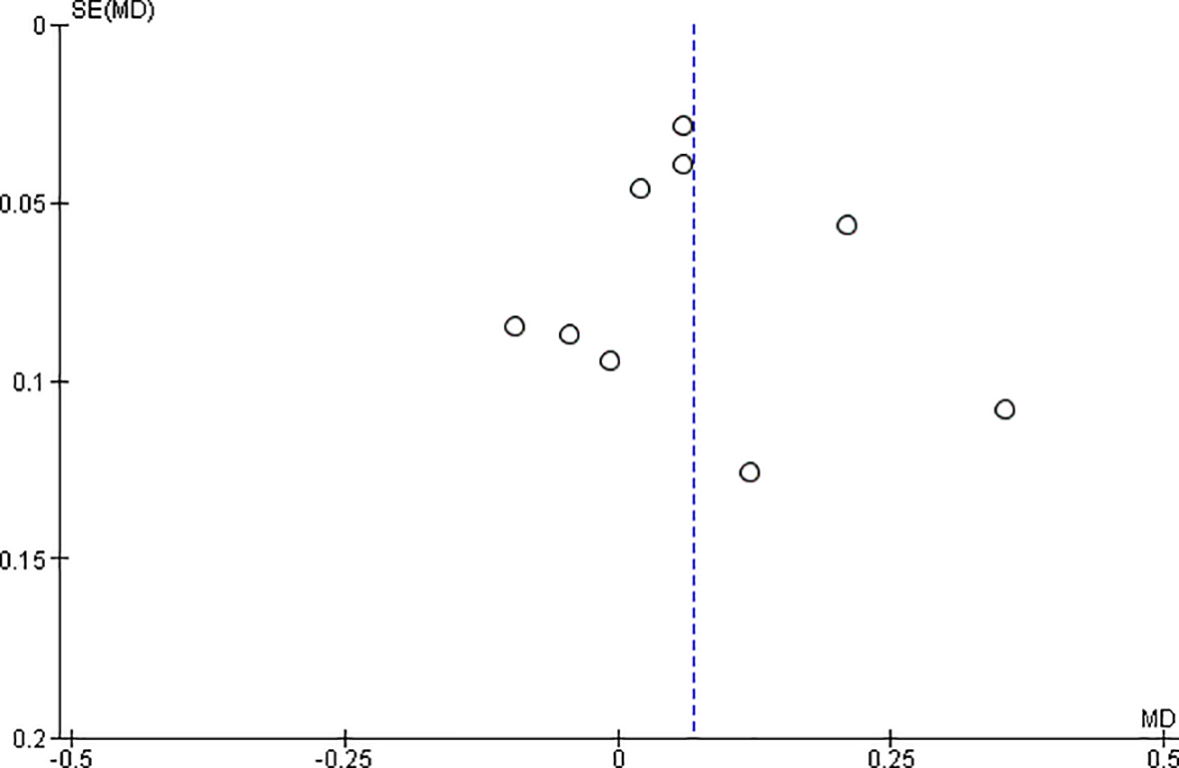
Funnel plot for surface roughness.

**Figure 6.**
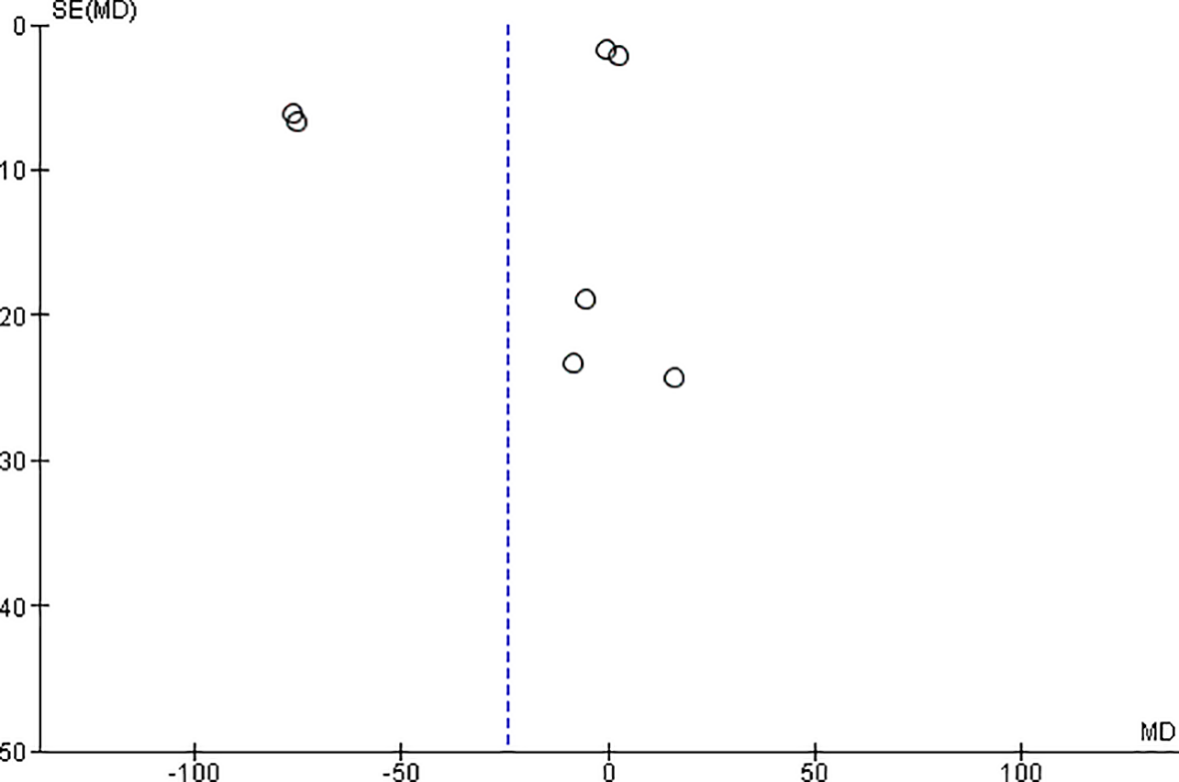
Funnel plot for microhardness.

The observed effect sizes were more or less symmetrically distributed around the combined effect size, in both the outcomes, indicating no asymmetry in the distribution of effect sizes and hence no evidence of publication bias ( Figures 5 & 6).

## Discussion

Whitening toothpastes are easily available over the counter without a prescription. The ingredients of a whitening paste range from abrasives such as hydrated silica, calcium carbonate to whitening agents like perlite, peroxides, activated charcoal, blue covarine, hydrogen peroxide and microbeads. ^1^ Ideally, whitening toothpastes must remove stains and improve tooth color. However, studies ^8^^,^^9^^,^^16^^,^^18^^,^^20^ have shown that whitening toothpastes also have deleterious effect on the mineral content of enamel resulting in increased surface roughness and reduced microhardness. This systematic review was carried out to examine the relationship between whitening toothpastes and surface roughness as well as microhardness of extracted human teeth.

Surface roughness and microhardness are important indications of loss or gain of mineral content in tooth structure which can be used to show the unwanted effect of whitening toothpastes. Among the 7 studies which were included in this systematic review, 5 studies ^8^^,^^9^^,^^16^^,^^18^^,^^20^ reported increase in surface roughness of enamel. However, in the study by Alpan *et al*, ^17^ among the 6 whitening toothpastes studied, two whitening toothpastes i.e., Splat Special Blackwood and Colgate Optic White, reduced enamel roughness. The study by Shamel *et al* ^19^ also showed that whitening toothpastes containing blue covarine produced less surface roughness compared to other whitening toothpastes. With respect to the other outcome of microhardness, two studies reported no effect of whitening toothpastes on microhardness ^16^^,^^20^ and two studies reported that whitening toothpastes reduced microhardness. ^8^^,^^9^

It is crucial to emphasize that the composition of the whitening toothpaste as well as the content and the type of whitening agent might affect the surface roughness and microhardness of the enamel surface. Moreover, the simulation of toothbrushing and the duration and frequency of its application were different in the 7 studies included for the systematic review, which could have influenced the outcomes.

When we analyzed the forest plot for surface roughness, we found that the meta-analytic effect is statistically significant with surface roughness value being higher in the intervention group i.e., the group which was administered the whitening toothpaste. When the forest plot for microhardness was analyzed, we found that the meta-analytic effect was statistically significant with the microhardness value being lesser in the intervention group.

Since the I ^2^ value was found to be 61% and 98% in the forest plot for surface roughness and microhardness indicating moderate and high heterogeneity respectively, the pooled statistics need to be interpreted with caution. However, it is also prudent to note that I ^2^ value is not a measure of absolute heterogeneity. Rather, it tells us what proportion of the observed variance reflects variance in true effect sizes rather than sampling error. ^21^

Since this meta-analysis is based on *in vitro* studies and we have also partitioned the data for analysis, the I ^2^ value may reflect the extent to which confidence intervals from the included studies overlap with each other.

### Limitations of the included evidence

All studies except one gave no information about the random allocation sequence, blinding of people delivering the intervention and blinding of the outcome assessors. Although these are *in vitro* studies, randomization plays a crucial role in minimizing bias. The studies included in this systematic review have used toothpastes containing different types of whitening agents such as hydrogen peroxide, charcoal and blue covarine. A few studies ^17^^,^^19^ have shown that some whitening agents produced less surface roughness when compared to other whitening agents. It is important that studies incorporate the exact whitening method used so that it can be correlated with the changes in surface roughness and microhardness.

## Conclusions

**Implications for practice:** Although whitening toothpastes typically can lighten tooth color by about one or two shades, there is some evidence to show that these toothpastes also affect the mineral content of teeth by increasing surface roughness and reducing microhardness. Therefore, dental professionals need to educate their patients to be cautious regarding the prolonged use of home use whitening toothpaste. ^22^

**Implications for policy:** More evidence and further research are needed to identify the type of whitening agent which will whiten the tooth effectively while maintaining the integrity of the tooth structure.

**Implications for future research:** This systematic review and meta-analysis has provided some evidence that whitening toothpastes do affect the surface roughness and microhardness of human teeth. However, further research with robust methodology, reducing the risk of bias, needs to be conducted to definitively establish the role of abrasive and whitening components in increasing the surface roughness and microhardness of human enamel.

### Registration and protocol

Since this was a systematic review and meta-analysis of *in vitro* studies, it could not be registered in PROSPERO. However, the review protocol can be found in the *Extended data*. ^23^

## Data availability

### Underlying data

All data underlying the results are available as part of the article and no additional source data are required.

### Extended data

Figshare: Effect of whitening toothpaste on surface roughness and micro hardness of human teeth - A systematic review and meta-analysis, https://doi.org/10.6084/m9.figshare.17128811 ^23^

The project contains the following extended data:

- Protocol.docx

### Reporting guidelines

Figshare: Effect of whitening toothpaste on surface roughness and micro hardness of human teeth - A systematic review and meta-analysis. https://doi.org/10.6084/m9.figshare.17128811 ^23^

The project contains the following reporting guidelines:

- PRISMA checklist.docx
- PRISMA flow chart.docx

Data are available under the terms of the Creative Commons Zero “No rights reserved” data waiver (CC0 1.0 Public domain dedication).
