# Supplementary material for: Effect of whitening toothpaste on surface roughness and microhardness of human teeth: a systematic review and meta-analysis
Source: F1000Res. 2022 Mar 8;11:22. Originally published 2022 Jan 11. [Version 3] doi: 10.12688/f1000research.76180.3 (PMC8874033; doi:10.12688/f1000research.76180.3)
Supplement: Peer review history

# Peer review: Reviewer response for version 3

**DOI:** 10.5256/f1000research.121868.r126618

**Published:** 2022-03-09

**Authors:** Harsh priya

**Affiliations:** 1 Division of Public Health Dentistry, Centre for Dental Education and Research, All India Institute of Medical Sciences, New Delhi, Delhi, India

All the suggestions addressed.

 Thank you.

 Best Wishes

Are the rationale for, and objectives of, the Systematic Review clearly stated?

Yes

Is the statistical analysis and its interpretation appropriate?

Partly

Are sufficient details of the methods and analysis provided to allow replication by others?

Yes

Are the conclusions drawn adequately supported by the results presented in the review?

Yes

Reviewer Expertise:

Dental Public Health

I confirm that I have read this submission and believe that I have an appropriate level of expertise to confirm that it is of an acceptable scientific standard.

# Peer review: Reviewer response for version 2

**DOI:** 10.5256/f1000research.121242.r119761

**Published:** 2022-02-21

**Authors:** Ashish Shrestha

**Affiliations:** 1 Department of Public Health Dentistry, College of Dental Surgery, B. P. Koirala Institute of Health Sciences, Dharan, Nepal

The review seems complete with the articles reviewed. Only articles written in English have been reviewed as per the expertise present. The authors have presented a full systematic review and explained the meta-analysis. The study selection as well of the risk of bias has been well explained. The review also contains limitations the authors faced while conducting the review. All in all it is appropriate and fit for indexing.

Are the rationale for, and objectives of, the Systematic Review clearly stated?

Yes

Is the statistical analysis and its interpretation appropriate?

Yes

Are sufficient details of the methods and analysis provided to allow replication by others?

Yes

Are the conclusions drawn adequately supported by the results presented in the review?

Yes

Reviewer Expertise:

Oral health, dental public health and preventive dentistry

I confirm that I have read this submission and believe that I have an appropriate level of expertise to confirm that it is of an acceptable scientific standard.

# Response

**Published:** 2022-02-22

**Authors:** Ashwini Rao

**Affiliations:** Manipal Academy of Higher Education, Manipal, India

Thank you

# Peer review: Reviewer response for version 1

**DOI:** 10.5256/f1000research.80145.r119769

**Published:** 2022-02-24

**Authors:** Harsh priya

**Affiliations:** 1 Division of Public Health Dentistry, Centre for Dental Education and Research, All India Institute of Medical Sciences, New Delhi, Delhi, India

Dear Authors,

 Congratulations on your manuscript.

 The systematic review and meta-analysis has defined the rationale and objective behind the conceptualization of this study. They have defined the in vitro randomized control trials that will be included in this review. The methods and analysis are appropriately defined for replication. The statistical analysis and interpretation of data is pertinent. The conclusion of the review that whitening toothpastes typically can lighten tooth colour by about one or two shades is for clinically application for the general public and private practitioners. There is some evidence generated to show that these toothpastes also affect the mineral content of teeth by increasing surface roughness and reducing microhardness. Such reviews can help clinicians and masses to decide on the continuous use of whitening toothpastes.

 Few minor comments are as below:

 Comment 1: Table 1- Characteristics of included studies

 Bolay 2012 - Exposed to 20.000 brush strokes

 (It should be 20,000)

 Rahardjo 2015 - Brushed for 840 seconds ti simulate 3 months.

 (It should probably be 840 minutes. If we calculate to simulate 5 minutes daily for 90 days it comes to 450 minutes, hence 840 seconds seem to be wrongly written)

 Alpan Lektemur 2020 - Brushed for 5 seconds per day for 30 days

 (5 seconds per day seems a little less. Need to cross check the data. Probably its 5 minutes per day)

 Comment 2:

 "7 studies that were included for systematic review, quantitative data was inappropriate for 3 studies and one showed high risk of bias and therefore these studies could not be included, and meta-analysis was conducted using the

 remaining 4 studies."

 Kindly recalculate. If 3 studies were inappropriate and 1 was not included due to high risk of bias. Seven minus four, i.e. three studies should have been included in meta-analysis.

 Hopefully this will improve the manuscript.

 All the best.

Are the rationale for, and objectives of, the Systematic Review clearly stated?

Yes

Is the statistical analysis and its interpretation appropriate?

Partly

Are sufficient details of the methods and analysis provided to allow replication by others?

Yes

Are the conclusions drawn adequately supported by the results presented in the review?

Yes

Reviewer Expertise:

Dental Public Health

I confirm that I have read this submission and believe that I have an appropriate level of expertise to confirm that it is of an acceptable scientific standard, however I have significant reservations, as outlined above.

# Response

**Published:** 2022-02-26

**Authors:** Ashwini Rao

**Affiliations:** Manipal Academy of Higher Education, Manipal, India

Thanks for the suggestions.

 The following suggestion was incorporated

 Comment 1: Table 1- Characteristics of included studies, Bolay 2012 - Exposed to 20.000 brush strokes (It should be 20,000) - Correction done.

 However, for the below comments, no changes were done since the article mentions 5 seconds and not 5 minutes.

 Comment 2: Rahardjo 2015 - Brushed for 840 seconds ti simulate 3 months. (It should probably be 840 minutes. If we calculate to simulate 5 minutes daily for 90 days it comes to 450 minutes, hence 840 seconds seem to be wrongly written)

 Response: No changes done:

 The article mentions brushing time as 5 seconds not minutes. So no changes were done.

 Comment 3: Alpan Lektemur 2020 - Brushed for 5 seconds per day for 30 days (5 seconds per day seems a little less. Need to cross check the data. Probably its 5 minutes per day)

 Response: No changes done:

 The article mentions brushing time as 5 seconds not minutes. So no changes were done

 Comment 4: "7 studies that were included for systematic review, quantitative data was inappropriate for 3 studies and one showed high risk of bias and therefore these studies could not be included, and meta-analysis was conducted using the remaining 4 studies." Kindly recalculate. If 3 studies were inappropriate and 1 was not included due to high risk of bias. Seven minus four, i.e. three studies should have been included in meta-analysis.

 Response: No changes done:

 One study (Shamel et al – Ref 19) had two issues, quantitative data was inappropriate and also showed high risk of bias. So, meta-analysis was conducted using the remaining 4 studies

 “Among the 7 studies that were included for systematic review, quantitative data was inappropriate for 3 studies (9 , 17 , 19) and one (19) showed high risk of bias and therefore these studies could not be included, and meta-analysis was conducted using the remaining 4 studies.”

# Peer review: Reviewer response for version 1

**DOI:** 10.5256/f1000research.80145.r119759

**Published:** 2022-02-10

**Authors:** Dimitrios Dionysopoulos

**Affiliations:** 1 Department of Operative Dentistry, Faculty of Dentistry, School of Health Sciences, Aristotle University of Thessaloniki, Thessaloniki, Greece

The current systematic review and meta-analysis is interesting and well conducted, albeit the data do not add any new information. It is crucial to be emphasize that the composition of the whitening toothpaste, as well as the content and the type of the whitening agent are the factors that mainly affect the surface roughness and microhardness of the enamel surface. Moreover, the simulation of toothbrushing and the duration and frequency of its application can influence those outcomes. It is also important to discuss that the effectiveness of this whitening method is correlated with the changes in surface roughness and microhardness.

Are the rationale for, and objectives of, the Systematic Review clearly stated?

Yes

Is the statistical analysis and its interpretation appropriate?

Yes

Are sufficient details of the methods and analysis provided to allow replication by others?

Yes

Are the conclusions drawn adequately supported by the results presented in the review?

Yes

Reviewer Expertise:

Operative Dentistry

I confirm that I have read this submission and believe that I have an appropriate level of expertise to confirm that it is of an acceptable scientific standard, however I have significant reservations, as outlined above.

# Response

**Published:** 2022-02-11

**Authors:** Ashwini Rao

**Affiliations:** Manipal Academy of Higher Education, Manipal, India

Dear sir,

 Thank you for the suggestions. We have incorporated the suggestions given:

1. In the discussion component, we have incorporated the role of the composition of the whitening toothpaste, as well as the content and the type of the whitening agent on the surface roughness and microhardness of the enamel surface, and also the influence of simulation of toothbrushing and the duration and frequency of its application on the outcomes.
2. In the limitation component, we have added a note on the importance of incorporating the exact whitening method used, in the studies, so that it can be correlated with the changes in surface roughness and microhardness.
